# Organization of Bone Sarcoma Care: A Cross‐Sectional European Study

**DOI:** 10.1111/os.12716

**Published:** 2020-06-26

**Authors:** Louren Matthias Goedhart, Andreas Leithner, Paul Christiaan Jutte

**Affiliations:** ^1^ Department of Orthopaedic surgery University of Groningen, University Medical Center Groningen Groningen The Netherlands; ^2^ Department of Orthopaedics and Trauma Medical University of Graz Graz Austria

**Keywords:** Bone sarcoma, Centralization, Organization of care

## Abstract

**Objective:**

To assess organization of care in several bone sarcoma centers in Europe affiliated with the European Musculoskeletal Oncology Society (EMSOS) for comparison and to identify potential improvements in organization of care.

**Methods:**

Data for this observational cross‐sectional study was obtained through healthcare professionals affiliated to EMSOS. The authors formulated 10 questions regarding organization of care. The questions were focused on guidance, multidisciplinary decision‐making, and data storage. A digital questionnaire was synthesized and included quality control. The digital questionnaire was sent to 54 representative members of EMSOS. We did not receive responses from 29 representative countries (53.7%) after one digital invitation and two digital reminders.

**Results:**

We received data from 25 representatives of bone sarcoma centers from 17 countries across Europe (46.3%). Authorization to perform oncological care in a bone sarcoma center was government issued in 41.2% of cases and based on expertise without governmental influence in 52.9% of cases. In 64.7% of the countries, a national bone tumor guideline regarding for diagnosis and treatment is used in oncological care. A national bone tumor board for extensive case evaluation including classification and advice for treatment is available for 47.1% of the countries. All participating bone sarcoma centers have a mandatory local multidisciplinary meeting before the start of treatment; in 84.0% this meeting takes place once a week. During this multidisciplinary meeting a median of 15 cases (range, 4–40 cases) are discussed. In terms of storage of oncological data, a local registry is used in eight countries (47.1%). A national registry is used in eight countries (47.1%).

**Conclusions:**

A national bone tumor board gives bone sarcoma centers with little adherence the opportunity to gain knowledge from a more experienced team. Centralization of care in a bone sarcoma center is important to lower incidences. The optimal size for a bone sarcoma center in terms of patient adherence is not known at present.

## Introduction

High‐grade bone sarcomas are aggressive tumors with a high potential of metastasis. Diagnosis and treatment of these neoplasms is challenging due to low incidences^1^, ^2^, ^3^, ^4^, ^5^.

Therefore, centralization of sarcoma care is important in order to realize a multidisciplinary approach by an experienced team^6^, ^7^. Nowadays, the majority of patients with a primary bone sarcoma are diagnosed and treated in a bone sarcoma center. A few dozen bone sarcoma centers with expertise are scattered across Europe. However, as for differences in nationwide organization of care, the approach towards diagnosis and treatment differs between these hospitals. Further differences are seen in terms of patient adherence to bone sarcoma centers due to centralization of care. Based on a single study, treatment in a bone sarcoma center was associated with higher survival for high‐grade osteosarcoma patients^8^. However, this association was not seen for high‐grade chondrosarcoma and Ewing sarcoma patients. Furthermore, the optimal size for a bone sarcoma center in terms of patient adherence is not known at this moment. The European Musculoskeletal Oncology Society (EMSOS) aims to promote advances in science, disseminate knowledge, and promote mutual collaboration for bone sarcoma care between the different affiliated bone sarcoma centers.

This study aims to assess organization of care in several bone sarcoma centers in Europe affiliated with EMSOS for comparison and improvement of knowledge.

## Methods

The European Musculoskeletal Oncology Society (EMSOS) was founded in 1987. The particular purpose of EMSOS is to facilitate a network for different specialists and institutes in order to improve treatment of musculoskeletal tumors. This is realized by collaborative research projects and disseminating knowledge through an annual congress.

**Figure 1:**
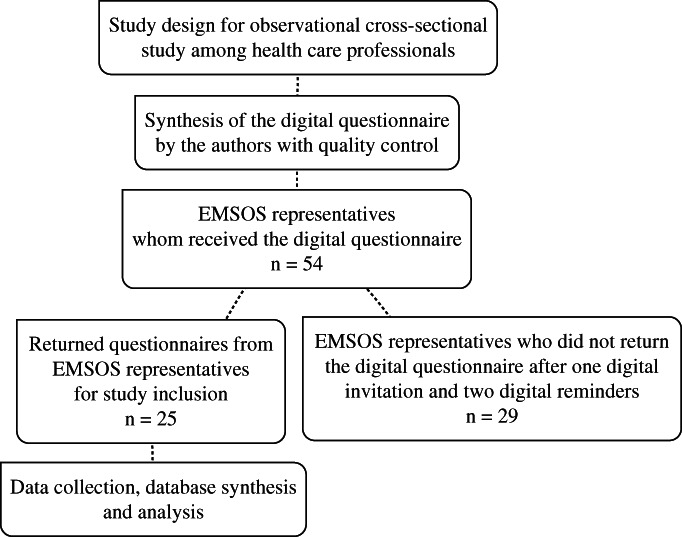
Flowchart of the study design.

Data for this observational cross‐sectional study was obtained through healthcare professionals affiliated with EMSOS. The authors formulated 10 questions regarding organization of care and produced a digital questionnaire, which is displayed in the appendix. The questions were focused on guidance, multidisciplinary decision‐making, and data storage. The digital questionnaire was not validated. EMSOS members were approached as representatives from all over Europe. These representatives were asked to return this digital questionnaire. A flowchart of the study design was displayed in Fig. 1. Observational research among healthcare professionals does not fall under the scope of the Dutch Act on Medical Scientific Research involving Human Beings (WMO).

Analyses were performed using IBM SPSS Statistics for Windows (Version 23.0, United States) and Microsoft Excel 2013 (United States).

## Results

**Figure 2:**
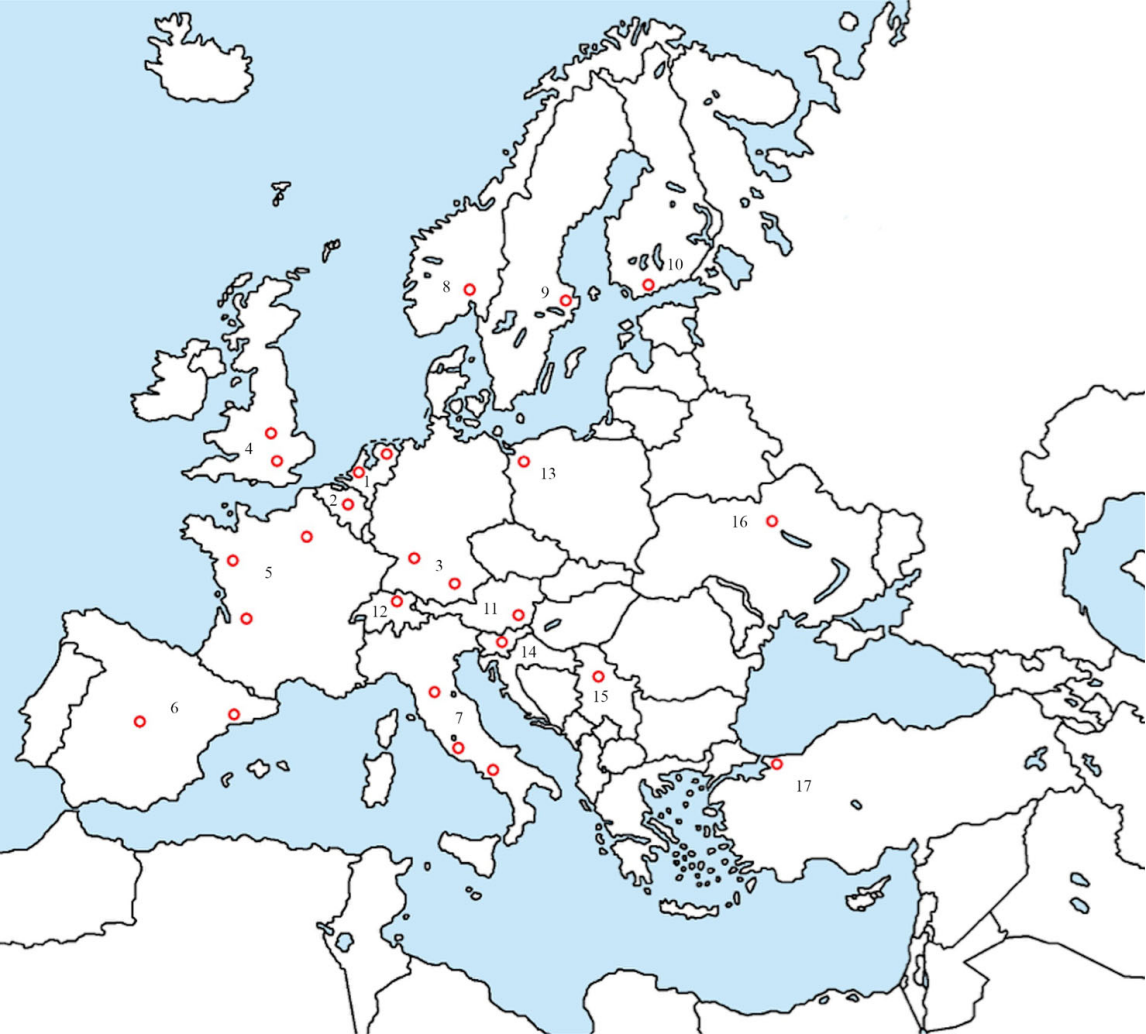
Geographical dispersion across Europe of responding bone sarcoma centres. 1. Netherlands: University Medical Center Groningen, Leiden University Medical Center. 2. Belgium: Jules Bordet Institute Brussels. 3. Germany: Medical Center of the University of Munich, Stuttgart Cancer Center Olgahospital. 4. United Kingdom: University College Hospital London, Royal Orthopedic Hospital Birmingham. 5. France: Limoges Teaching Hospital, University Hospital Hotel‐Dieu Nantes, Hospital Cochin Paris. 6. Spain: Hospital Universitario de Bellvitge Barcelona, Hospital Universitario La Paz Madrid. 7. Italy: Centro Traumatologico Ortopedico Florence, Regina Elena National Cancer Institute Rome, Cancer Institute G. Pascal Foundation Naples. 8. Norway: Oslo University Hospital. 9. Sweden: Karolinska Hospital Stockholm. 10. Finland: Helsinki University Central Hospital. 11. Austria: Medical University of Graz. 12. Switzerland: Balgrist University Hospital Zürich. 13. Poland: Pomeranian Medical University of Szczecin. 14. Slovenia: Ljubljana University Medical Centre. 15. Serbia: Institute for Oncology and Radiology Belgrade. 16. Ukraine: National Cancer Institute Kiev. 17. Turkey: Acibadem Maslak Hospital Istanbul.

A digital questionnaire was sent to 54 representative members of EMSOS and we received a response from 25 representatives (46.3%) from 17 countries after one digital invitation and two digital reminders. These representatives were acknowledged as the EMSOS study group. The geographical dispersion across Europe of responding bone sarcoma centers was displayed in Fig. 2. Questionnaire output data regarding bone sarcoma centers per country were displayed in Table 1.

**Table 1:** Questionnaire output data regarding bone sarcoma centres per country

	Number of bone sarcoma centres	Million inhabitants in 2018^9^	Million inhabitants per bone sarcoma centre	Authorisation basis	Bone tumor guideline	National bone tumor board	Number of cases discussed per week in local meeting	Referral to a bone sarcoma centre (%)	Treatment in a bone sarcoma centre (%)	Registry for oncological data
Netherlands	4	17.15	4.28	Government	National	Yes	25–30	90	98	National
Belgium	5	11.57	2.31	Expertise	Local	No	12	50	90	Local
Germany	Not clear*	80.46	Not clear*	Not clear*	National	No	5–20	Not clear*	Not clear*	Local†
United Kingdom	5	65.11	13.02	Government	National	Yes	40	90	100	National
France	12	67.36	5.61	Expertise	National	Yes	15–40	90	98	National
Spain	10	49.33	4.93	Expertise	National	No	6–10	70	30	Local
Italy	10	62.25	6.22	Expertise	Local	No	10–15	90	95	Local
Norway	2	5.37	2.69	Government	National	No	10	90	100	Local
Sweden	3	10.04	3.35	Government	National	Yes	20	95	95	National
Finland	4	5.54	1.38	Government	Local	Yes	25	90	99	Local
Austria	4	8.79	2.19	Expertise	Local	No	15	70	90	National
Switzerland	5	8.29	1.66	Expertise	National	No	12	95	N/A	Local
Poland	5	38.42	7.68	Government	National	Yes	4	70	80	National
Slovenia	1	2.10	2.10	Expertise	National	Yes	12	N/A	95	National
Serbia	1	7.08	7.08	Expertise	Local	Yes	10	70	80	Local
Ukraine	4	43.95	10.98	Government	National	No	4	50	30	National
Turkey	5	81.26	16.25	Expertise	Local	No	20	5	20	No registry

N/A, not available

* In Germany bone sarcoma centres are not defined, therefore it is not clear where bone sarcoma patients are treated in this country

† Cooperative German‐Austrian‐Swiss Osteosarcoma Study Group (COSS) and Cooperative German‐Austrian‐Swiss Ewing sarcoma Study Group (CESS) are multinational initiatives for registration of oncological data.

### *Guidance*

Authorization to perform oncological care in a bone sarcoma center was government issued in the Netherlands, the United Kingdom, Norway, Sweden, Finland, Poland, and Ukraine (41.2%). Authorization based on expertise without governmental influence was seen in Belgium, France, Spain, Italy, Austria, Switzerland, Slovenia, Serbia, and Turkey (52.9%). A lack of consensus towards authorization of bone sarcoma centers was seen in Germany, there are not a defined number of bone sarcoma centers in this country. In 64.7% of the countries, a national bone tumor guideline regarding diagnosis and treatment is used in oncological care. In Belgium, Italy, Finland, Austria, Serbia, and Turkey, local hospital guidelines are used for diagnosis and treatment (35.3%). Several (national) bone tumor guidelines, obtained through the questionnaire, are displayed in the appendix.

### *Multidisciplinary Decision Making*

A national bone tumor board for extensive case evaluation including classification and advice for treatment is available in the Netherlands, the United Kingdom, France, Sweden, Finland, Poland, Slovenia, and Serbia (47.1%). All participating bone sarcoma centers have a mandatory local multidisciplinary meeting before the start of treatment; in the vast majority this meeting takes place once a week (84.0%). During this multidisciplinary meeting a median of 15 cases (range, 4–40 cases) are discussed. Regarding referral towards and treatment in a bone sarcoma center, most countries had percentages in the upper quartiles as shown in Table 1. Lower referral percentages were seen in Belgium (50%), Ukraine (50%), and Turkey (5%). With regards to treatment in a bone sarcoma center, relatively low treatment percentages were seen in Spain (30%), Ukraine (30%), and Turkey (20%).

### *Data storage*

A local registry for oncological data is used in Belgium, Germany, Spain, Italy, Norway, Finland, Switzerland, and Serbia (47.1%). A national registry is used in the Netherlands, the United Kingdom, France, Sweden, Austria, Poland, Slovenia, and Ukraine (47.1%). Bone tumors are not registered in Turkey (5.8%).

## Discussion

Dedicated health care professionals all over Europe perform bone sarcoma care. This is the first study to provide cross‐sectional data regarding organization of bone sarcoma care in Europe. A wide range of centralization across Europe was identified. Limitations of this study are clear because of the observational concept. Furthermore, the questionnaire we used was not validated. Finally, although the respondents represent a large proportion of Europe, the response rate of 46.3% could have led to response bias.

The basis on which oncological care in a bone sarcoma center is performed differs.

Most bone sarcoma centers are authorized based on expertise, and government authorization has been issued in the countries where the government has extensive responsibilities for national health care. In a considerable number of countries bone tumor guidelines are issued for diagnostic work‐up, referral, and treatment. We believe that these guidelines are a valuable instrument for the clinicians. A recent development is that the European Commission launched an initiative for European Reference Networks (ERN) to create a network of excellence in clinical practice. These networks aim to facilitate discussion on and improve care of complex or rare diseases^10^. Furthermore, essential requirements for quality cancer care for soft‐tissue and bone sarcoma in adults were defined by the European CanCer Organization (ECCO)^11^. Partially based on these developments, a survey among Italian oncological health care professionals resulted in a set of minimum requirements needed to define a referral center for rare cancers^12^.

An interesting finding from our study is the lack of consensus towards authorization of bone sarcoma centers in Germany, as shown in Table 1. Germany is clearly different from the other countries regarding its organization and centralization. Until now, a definition of a bone sarcoma center has never been developed in Germany, resulting in decentralization of bone sarcoma care towards treatment centers.

Decentralization of bone sarcoma care in a country could have adverse effects in terms of delay in diagnosis, misdiagnosis, and inappropriate treatment. Delay in diagnosis in high‐grade bone sarcomas from symptoms until the start of the treatment has been described in the literature^13^. Delay is inevitable, but minimizing delay using clear guidelines and referral patterns seems judicious. As mentioned earlier, assessment of radiology and histology by an experienced team is essential for a good prognosis in chondrosarcoma^7^. Furthermore, misdiagnosis and subsequent inappropriate treatment resulted in inferior outcomes in osteosarcoma^14^. For Ewing sarcoma, inadequate surgical margins are significantly correlated with inferior outcome^15^. A study regarding soft‐tissue sarcoma concluded that patients treated in high‐volume hospitals less often had macroscopic residual disease^6^.

At an earlier stage, comprehensive incidence estimates were published for all the main primary bone sarcomas in the Netherlands^8^. These incidences for high‐grade chondrosarcoma (0.15 per 100,000 European Standardized Rate (ESR)), high‐grade central osteosarcoma (0.25 per 100,000 ESR), and Ewing sarcoma (0.15 per 100,000 ESR) are relatively low compared to other cancer types.

We believe that centralization of care towards a bone sarcoma center is sensible given these incidences, regardless of the basis of authorization or government inference.

In our study, we reproduced the availability of a bone sarcoma center for bone sarcoma patients based on the number of inhabitants of the represented country. A major increase in the adherence per bone sarcoma center could result in a low referral and treatment percentage, with Turkey and Ukraine as an example as shown in Table 1. A possible explanation could be the increased geographical dispersion in the less populated areas of these big countries.

The ECCO expert group recommends that at least 50 bone sarcoma patients are treated in a bone sarcoma center every year^11^. This threshold is based on guidance from the British National Institute for Health and Care Excellence (NICE)^16^. Conversely, the authors state that this threshold of bone sarcoma in 50 patients every year is dependent on referral patterns and expertise distribution. Bone sarcoma patients are defined by the ECCO as patients with chondrosarcoma, Ewing sarcoma, and osteosarcoma. Furthermore, very rare entities as undifferentiated bone sarcoma, chordoma, and giant cell tumor of bone are defined as bone sarcomas by the ECCO^11^. Interestingly, the actual exposure of a bone sarcoma center could be calculated based on our data. A calculation could be made in which the combined incidence for high‐grade chondrosarcoma, high‐grade central osteosarcoma, and Ewing sarcoma (0.55 per 100,000 ESR) is matched with a minimum exposure of 50 bone sarcoma patients for a single bone sarcoma center every year. This is roughly four patients per month. Based on the ECCO recommendation, a single bone sarcoma center should have a minimal adherence of around 9 mn inhabitants to match the exposure of 50 bone sarcoma patients. Based on our study, this exposure can only be matched by bone sarcoma centers in the United Kingdom, Turkey, and Ukraine. However, as mentioned earlier regarding Turkey and Ukraine, more inhabitants per bone sarcoma center could result in a low referral and treatment percentage of bone sarcoma patients, which seems undesirable. Based on our study, the effects of centralization could not be assessed. Therefore, the optimal size for a bone sarcoma center is not known at present. We believe that the participating bone tumors centers in our study provide excellent bone sarcoma care. The recommendation of 50 bone sarcoma patients per year is based on existing evidence as stated in the ECCO article with a reference to the 2006 NICE guidance document^11^, ^16^. In this guidance document, the authors refer to studies from the United Kingdom and Sweden, which conclude that treatment of a bone sarcoma in a specialist center is preferred, without notice of a minimum threshold for treatment per year^17^, ^18^. This suggests that the treatment threshold for a bone sarcoma center of 50 bone sarcoma patients per year is not evidence‐based. We believe that the treatment threshold for a bone sarcoma center per year for adequate treatment of their patients is not known at this moment. To evaluate this, a comparative study between differently sized bone sarcoma centers regarding survival in high‐grade bone sarcoma patients could be a next step. This should give more clarity about the actual effect of centralization of care on survival. Although the treatment threshold is not known, we think that a minimum treatment threshold of at least one bone sarcoma patient every month in a single bone sarcoma center is desirable. This preserves the available expertise of the multidisciplinary team. Reasonably, bone sarcoma centers with little adherence could benefit from a national bone tumor board for extensive case evaluation including classification and advice for treatment from a team with more experience.

A national registry is the basis for adequate monitoring and reporting of outcomes. Furthermore, a complete national registry could provide valuable and comparative big data for collaborative research, which is needed with the given low incidences for high‐grade bone sarcomas. This is emphasized by the previously published collaborative EMSOS studies for several rare entities^19^, ^20^, ^21^, ^22^. In our study, the effect of evaluation of care using a national registry was not investigated. Still we think that better evaluation of care, as one can do with a registry, provides essential information to improve quality of care and outcome for bone sarcoma patients.

In conclusion, we believe that centralization of care towards a bone sarcoma center should be mandatory. The optimal size for a bone sarcoma center in terms of patient adherence is not known at this moment. Furthermore, a national registry is essential for the adequate storage and reproduction of oncological data.

**EMSOS Study Group**
Michiel van der Sande: Leiden University Medical Center, the Netherlands
Félix Shumelinsky: Jules Bordet Institute Brussels, Belgium
Minna Laitinen: multiple myeloma
PBSC: Helsinki University Central Hospital, Finland
Fabrice Fiorenza: Limoges Teaching Hospital, France
Domenico Campanacci: Centro Traumatologico Ortopedico Florence, Italy
Federico Portabella: Hospital Universitario de Bellvitge Barcelona, Spain
Henrik Bauer: Karolinska Hospital Stockholm, Sweden
Emre Aycan: Acibadem Maslak Hospital Istanbul, Turkey
Bruno Fuchs: Balgrist University Hospital Zürich, Switzerland
Anatolii Diedkov: National Cancer Institute Kiev, Ukraine
Carmine Zoccali: Regina Elena National Cancer Institute Rome, Italy
Hans Roland Dürr: Medical Center of the University of Munich, Germany
Eduardo Ortiz‐Cruz: Hospital Universitario La Paz Madrid, Spain
Flavio Fazioli: Cancer Institute G. Pascal Foundation Naples, Italy
François Gouin: University Hospital Hotel‐Dieu Nantes, France
Stefan Bielack: Stuttgart Cancer Center Olgahospital, Germany
Ole‐Jacob Norum: Oslo University Hospital, Norway
Jelena Bokun: Institute for Oncology and Radiology, Belgrade, Serbia
Blaz Mavcic: Ljubljana University Medical Centre, Slovenia
Daniel Kotrych: Pomeranian Medical University of Szczecin, Poland
Lee Jeys: Royal Orthopedic Hospital Birmingham, United Kingdom
Jeremy Whelan: University College Hospital London, United Kingdom
David Biau: Hospital Cochin Paris, France

## Authorship Declaration

The authorship criteria according to the latest guidelines of the International Committee of Medical Journal Editors are met by the authors. All are in agreement with the manuscript.

## References

[1] van Oosterwijk JG , Anninga JK , Gelderblom H , Cleton‐Jansen AM , Bovee JV . Update on targets and novel treatment options for high‐grade osteosarcoma and chondrosarcoma. Hematol Oncol Clin North Am, 2013, 27: 1021–1048. 24093174 10.1016/j.hoc.2013.07.012

[2] Anninga JK , Gelderblom H , Fiocco M , *et al* Chemotherapeutic adjuvant treatment for osteosarcoma: where do we stand? Eur J Cancer, 2011, 47: 2431–2445. 21703851 10.1016/j.ejca.2011.05.030

[3] Duchman KR , Gao Y , Miller BJ . Prognostic factors for survival in patients with Ewing's sarcoma using the surveillance, epidemiology, and end results (SEER) program database. Cancer Epidemiol, 2015 Apr, 39: 189–195. 25595632 10.1016/j.canep.2014.12.012

[4] Duchman KR , Gao Y , Miller BJ . Prognostic factors for survival in patients with high‐grade osteosarcoma using the surveillance, epidemiology, and end results (SEER) program database. Cancer Epidemiol, 2015, 39: 593–599. 26002013 10.1016/j.canep.2015.05.001

[5] Allison DC , Carney SC , Ahlmann ER , *et al* A meta‐analysis of osteosarcoma outcomes in the modern medical era. Sarcoma, 2012, 2012: 704872. 22550423 10.1155/2012/704872 PMC3329715

[6] Hoekstra HJ , Haas RLM , Verhoef C , *et al* Adherence to guidelines for adult (non‐GIST) soft tissue sarcoma in The Netherlands: a plea for dedicated sarcoma centers. Ann Surg Oncol, 2017, 24: 3279–3288. 28748443 10.1245/s10434-017-6003-3 PMC5596052

[7] Gelderblom H , Hogendoorn PC , Dijkstra SD , *et al* The clinical approach towards chondrosarcoma. Oncologist, 2008, 13: 320–329. 18378543 10.1634/theoncologist.2007-0237

[8] Goedhart LM , Ho VKY , Dijkstra SPDS , *et al* Bone sarcoma incidence in the Netherlands. Cancer Epidemiol, 2019, 60: 31–38. 30903831 10.1016/j.canep.2019.03.002

[9] CIA. The world factbook [Internet]. Available from: https://www.cia.gov/library/publications/the-world-factbook/.

[10] European Commission . European Reference Networks [Internet]. Available from: https://ec.europa.eu/health/ern_en.

[11] Andritsch E , Beishon M , Bielack S , *et al* ECCO essential requirements for quality cancer care: soft tissue sarcoma in adults and bone sarcoma. A critical review. Crit Rev Oncol Hematol, 2017, 110: 94–105. 10.1016/j.critrevonc.2016.12.002. 28109409

[12] Gronchi A , Delrio P , Quagliuolo V , Sandrucci S . The Italian Society of Surgical Oncology (SICO) survey on the minimum requirements of rare cancers referral centers. Updates Surg, 2016, 68: 321–323. 27312931 10.1007/s13304-016-0376-x

[13] Goedhart LM , Gerbers JG , Ploegmakers JJW , Jutte PC . Delay in diagnosis and its effect on clinical outcome in high‐grade sarcoma of bone: a referral oncological centre study. Orthop Surg, 2016, 8: 122–128. 27384720 10.1111/os.12239 PMC6584288

[14] Kim MS , Lee SY , Cho WH , *et al* Prognostic effects of doctor‐associated diagnostic delays in osteosarcoma. Arch Orthop Trauma Surg, 2009, 129: 1421–1425. 19280203 10.1007/s00402-009-0851-7

[15] Ozaki T , Hillmann A , Hoffmann C , *et al* Significance of surgical margin on the prognosis of patients with Ewing's sarcoma: a report from the cooperative Ewing's sarcoma study. Cancer, 1996, 78: 892–900. 8756387 10.1002/(SICI)1097-0142(19960815)78:4<892::AID-CNCR29>3.0.CO;2-P

[16] NICE Guidance . Improving outcomes for people with sarcoma [Internet]. Available from: https://www.nice.org.uk/guidance/csg9/evidence/full-guideline-pdf-2188960813.

[17] Stiller CA , Passmore SJ , Kroll ME , Brownbill PA , Wallis JC , Craft AW . Patterns of care and survival for patients aged under 40 years with bone sarcoma in Britain, 1980–1994. Br J Cancer, 2006, 94: 22–29. Available from: http://www.nature.com/doifinder/10.1038/sj.bjc.6602885. (accessed 2 April 2017). 16317433 10.1038/sj.bjc.6602885 PMC2361067

[18] Bergh P , Gunterberg B , Meis‐Kindblom JM , Kindblom LG . Prognostic factors and outcome of pelvic, sacral, and spinal chondrosarcomas: a center‐based study of 69 cases. Cancer, 2001;91 :1201–1212. 11283918 10.1002/1097-0142(20010401)91:7<1201::aid-cncr1120>3.0.co;2-w

[19] Grimer RJ , Gosheger G , Taminiau A , *et al* Dedifferentiated chondrosarcoma: prognostic factors and outcome from a European group. Eur J Cancer, 2007, 43: 2060–2065. 17720491 10.1016/j.ejca.2007.06.016

[20] Verdegaal SHM , Bovee JVMG , Pansuriya TC , *et al* Incidence, predictive factors, and prognosis of Chondrosarcoma in patients with Ollier disease and Maffucci syndrome: an international multicenter study of 161 patients. Oncologist, 2011, 16: 1771–1779. Available from: http://theoncologist.alphamedpress.org/cgi/doi/10.1634/theoncologist.2011-0200. 22147000 10.1634/theoncologist.2011-0200 PMC3248776

[21] Frezza AM , Cesari M , Baumhoer D , *et al* Mesenchymal chondrosarcoma: prognostic factors and outcome in 113 patients. A European musculoskeletal oncology society study. Eur J Cancer, 2015, 51: 374–381. 25529371 10.1016/j.ejca.2014.11.007

[22] Longhi A , Bielack SS , Grimer R , *et al* Extraskeletal osteosarcoma: a European Musculoskeletal Oncology Society study on 266 patients. Eur J Cancer, 2017, 74: 9–16. 10.1016/j.ejca.2016.12.016. 28167373

